# Role of TNF α, IL-6 and CXCL10 in Dengue disease severity

**Published:** 2018-06

**Authors:** Kiran Iqbal Masood, Bushra Jamil, Maryam Rahim, Muniba Islam, Muhammad Farhan, Zahra Hasan

**Affiliations:** Department of Pathology and Laboratory Medicine, The Aga Khan University, Karachi, Pakistan

**Keywords:** Dengue, IL6, CXCL10

## Abstract

**Background and Objectives:**

Dengue virus infections (Dengue) have become increasingly common in Pakistan and can result in case fatalities if not managed appropriately. Patients with Dengue virus infection may be asymptomatic or present with Dengue fever (DF), Dengue with warning signs (DWS) or severe Dengue (SD). Severity in Dengue is coincident with an exacerbated production of lymphocyte-induced cytokines and chemokines which are associated with plasma leakage. We investigated the association of circulating levels of cytokines such as Interleukin (IL)-6, tumor necrosis factor (TNF)-alpha and CXCL-10 in Dengue patients with differing severity of disease.

**Materials and Methods:**

Dengue infection was confirmed by testing for human IgM to the Dengue virus. Dengue patients (n=58) and healthy controls (n=33) were recruited. Dengue patients were grouped into those with DF (n=39), DWS (n=15) and SD (n=4). Serum IL-6, TNFα and CXCL10 levels were tested by ELISA. The Mann Whitney U test was used for statistical analysis.

**Results:**

Circulating levels of TNFα (p≤0.001) and CXCL10 (p≤0.001) levels were increased in Dengue patients as compared with controls. When patients were stratified for disease severity, it was observed that CXCL10 was increased in DWS as compared to DF (p=0.046). IL-6 levels were increased in patients with SD as compared to those with DWS (p=0.044). TNFα levels were not found to differ between different groups of Dengue patients.

**Conclusion:**

Raised CXCL10 and TNFα levels were associated with increased clinical severity of Dengue infection and probably increased disease progression due to excessive inflammation and increased vascular changes in the patients.

## INTRODUCTION

Dengue infection caused by the Dengue virus (DENV) of the Flaviviridae family (1) is a mosquito-borne disease which poses a significant global burden, infecting between 50–100 million people per year (2). The virus is rampant in tropical and sub-tropical regions, where the climate favors the rapid growth of the mosquito vector. A variety of factors including overpopulation, poor socio-economic conditions, lack of public health awareness and excessive flooding have led to the rapid spread of the disease (3). Dengue is now a serious public health concern in Pakistan and 21,597 positive cases were reported in 2015 (4).

Dengue infections have different manifestations which are classified as per WHO criteria into mild asymptomatic fever (DF), Dengue with warning signs (DWS) and severe Dengue (SD) (5). DF is defined as an acute fever with at least two of the following symptoms: nausea, vomiting, rash, aches and pains, tourniquet test positive and leukopenia. DWS is characterized by symptoms such as abdominal pain or tenderness, persistent vomiting, mucosal bleeding, liver enlargement >2 cm, increase in Hematocrit (HCT) concurrent with rapid decrease in platelet count. Criteria for SD is severe plasma leakage leading to shock and/or fluid accumulation with respiratory distress; severe bleeding as evaluated by clinician; severe organ involvement: with raised liver enzymes Aspartate aminotransferase (AST) or Ala-nine aminotransferase (ALT) ≥ 1,000, together with central nervous system (CNS) impairment (5).

Dengue primarily targets mononuclear cells within the leucocyte system. Cells infected with the Dengue virus produce nitric acid and protective pro-inflammatory cytokines such as Interferon-gamma (IFN-γ), Tumour Necrosis factor-alpha (TNF-α), interleukin-6 (IL-6) and IL-10 to attract and activate leucocytes at the site of infection (6). TNF-α is a pro-inflammatory cytokine produced by T cells, monocytes and endothelial cells to mediates a type-1 T cell response (7). IL-6 is a pro-inflammatory, pleiotropic cytokine that up-regulates inflammatory responses in macrophages and induces B cell maturation (8). IL-6 is also a pyrogen and induces fever in patients with dengue. CXCL-10 is an IFN-inducible chemokine, a chemo-attractant that plays a role in the innate immune response. CXCL10 binds CXCR3, a receptor expressed on lymphocytes, and in Dengue infections, CXCL10 recruits activated T and NK cells to the site of infection (9).

The relationship between cytokine activation in the host in response to the pathogen in Dengue infection is complex and makes it difficult to gauge severity of disease. Previous reports have identified a role of IL-6, TNF-α and CXCL10 in the progression of disease in Dengue infections, however, data showing the association of these cytokines with SD is limited. We investigated the association of the cytokines TNFα, IL-6 and CXCL10 in patients with differing Dengue disease severity as compared with healthy controls. This would allow us to identify markers of increased disease progression in Dengue infections.

## MATERIALS AND METHODS

### Subject selection.

This was a hospital based case control study. Dengue virus positive patients diagnosed at the Aga Khan University Hospital (AKUH) Clinical Laboratory, Karachi were included in the study. This cohort has been described previously with ethical approval from the Ethical Review Committee of the Aga Khan University, Pakistan (10). Here we focused on cases from a subset of Dengue patients (n=58) from a previous study (10) who were in-patients at the AKUH. Retrospective review of patient hospital and laboratory records was conducted. Patients with Dengue infection were categorized into Dengue fever (DF), Dengue with warning signs (DWS) and severe Dengue (SD) according to the 2009 WHO guidelines (5). For comparison, healthy controls (n=33) were also included in the study.

### Dengue IgM ELISA.

Serum samples were tested using Panbio Dengue IgM Capture ELISA kit as per manufacturer’s instruction (Panbio, Australia).

### CXCL10, TNF-α and IL-6 measurement.

Serum samples were used to measure CXCL10, TNF-α, and IL-6 levels. ELISA reagents and monoclonal antibodies for CXCL10 were obtained from R&D Systems (Abingdon, UK), for TNF-α were obtained from Thermoscientific (USA) and for IL-6 were obtained from Endogen (Rockford, IL, USA). All measurements were carried out according to the manufacturer’s recommendations. Recombinant human cytokine was used to obtain a dose response curve with a range of detection from 6.25–500 pg/ml for CXCL10, 4.4–1000 pg/ml for TNFα and 6.25–1000 pg/ml for IL-6. Readings for all cytokines were taken at 450 nm and were read off a standard curve.

### Statistical analysis.

Data are depicted as median values for each group with the IQR (inter quartile range 25th–75th percentile) indicated in each case. Comparison of non-parametric data between two groups was performed using the Mann–Whitney U-test. Analysis was performed and data plotted using GRAPHPAD PRISM Version 5 (GraphPad Software, San Diego, CA, USA).

## RESULTS

### Characteristics of the study group.

A total of 58 patients with Dengue infection were included in this study and they comprised fifteen cases of DF, thirty nine cases of DWS and four cases of SD. For comparison, 33 healthy controls who were age and sex matched were also included in the study. The characteristics of the study population are summarized in Table 1. Platelet count of study subjects with DF (p<0.001), DWS (p<0.001) and SD (p=0.0016) were significantly decreased as compared with healthy controls (Table 1).

**Table 1.** Characteristics of Dengue patients and healthy controls

**Characteristic**	**HC**	**DF (n= 15)**	**DWS (n= 39)**	**SD (n= 4)**
Age, mean ± SD, years	33±11	24.3 ± 16	39.9 ± 24.4	37 ± 27.3
Sex, Male (%)	M: 8 (53)	9 (60)	30 (77)	3 (75)
Female (%)	F: 7 (47)	6 (40)	9 (23)	1 (25)
Platelet count, mean ± SD error, platelets/mm^3^	279 ±16.2	156.2 ± 15.8*	69.9 ± 15.9*	35.3 ± 14.1*

HC, healthy controls (n=33); Cases with DF, Dengue fever (n=15); DWS, Dengue with warning signs (n=39); SD, Severe Dengue (n=4). Significant differences (p≤0.05) were calculated between the groups using Mann Whitney U test.

### Increased CXCL10 and TNFα levels in patients with Dengue as compared with healthy controls.

**Fig. 1.**
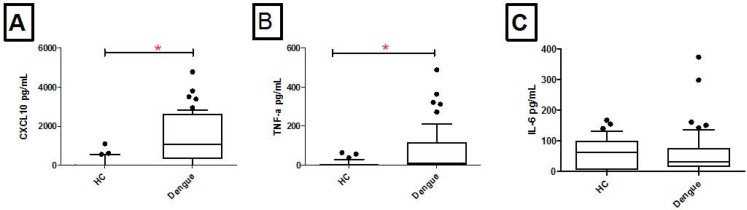
CXCL10, TNFα and IL-6 levels in patients with Dengue and healthy controls. Serum was collected from healthy controls (HC, 33) and Dengue patients (n=58) and subjected to ELISA to measure CXCL10, TNFα and IL6. Graphs show median values with standard deviation as y error bars. Data is shown for healthy controls and patients with Dengue for a) CXCL10, b) TNFα and c) IL-6 secretion levels. Level of significance was defined as P ≤ 0.05. Mann Whitney U test was used to compare the difference between the two groups.

Patients with Dengue had raised levels of CXCL10 (P <0.001) and TNFα (P <0.001) as compared with healthy controls (Fig. 1). However, IL-6 levels were found to be comparable between Dengue patients as compared with controls. To further investigate differential secretion of cytokines as per severity of clinical Dengue infections, we compared responses between patients across the clinical spectrum of dengue.

### Increased CXCL10 and TNFα are associated with increased disease severity in Dengue patients.

**Fig. 2.**
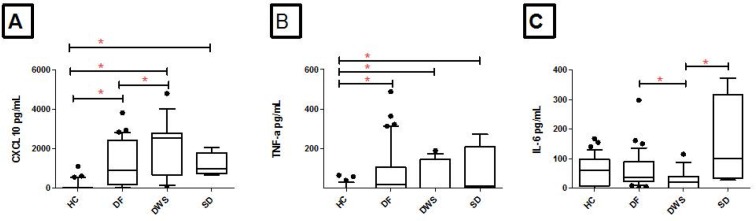
CXCL10, TNFα and IL-6 levels in patients with Dengue patients with increasing disease severity and healthy endemic controls. Serum was collected from healthy controls (HC, 33) and Dengue patients categorized into Dengue fever (DF, n=15), Dengue with warning signs (DWS, n=39) and Severe Dengue (SD, n=4) and subject to ELISA for CXCL10, TNFα and IL6. Graphs show medians values with standard deviation as y error bars. Data is shown for healthy controls and DF, DHF and DSS for a) CXCL10, b) TNFα and c) IL-6 secretion levels. Level of significance was defined as P ≤ 0.05. Mann Whitney U test was used to compare the difference between the two groups.

We determined CXCL10, IL-6 and TNFα levels in Dengue patients categorized into DF, DWS and SD. This data was also compared with the healthy control group (HC). CXCL10 secretion levels were increased in each of the Dengue groups; DF (P <0.001), DWS (P <0.001) and SD (P <0.001) as compared with healthy controls. When comparison was made between Dengue groups, CXCL10 levels were also found to be raised in DWS as compared with DF (P=0.046) (Fig. 2A). TNFα secretion levels were increased in serum of individual Dengue groups; DF (P <0.001), DWS (P=0.005) and SD (P=0.004) as compared with healthy controls (Fig. 2B). There was no difference in circulating levels of TNFα between patients with DF, DWS or SD. When serum IL-6 levels were compared between HC and different Dengue groups, it was found that IL-6 levels were similar (Fig. 2C). However, within Dengue disease severity groups, IL-6 levels were decreased in DWS as compared with DF (P=0.026) as well as SD (p=0.049).

## DISCUSSION

While up-regulation of immune responses, especially cytokine activation, is essential to control viral replication, an excess of cytokines results in increased inflammation and disease progression. Previous reports have shown that there is an increased profile of pro-inflammatory cytokines in patients with Dengue fever and Dengue hemorrhagic fever or Dengue with warning signs have been reported, however, data for severe Dengue is scanty. We observed that TNFα and CXCL10 levels were raised in patients with Dengue as compared with healthy controls. We observed varying circulating cytokine levels in Dengue patients depending on the severity of their disease. Particularly, we observed that circulating levels of TNFα and CXCL10 differed between Dengue patients with severe as compared with mild disease.

DENV can manipulate host mechanisms for its own survival and can switch protective Th1 responses to anti-inflammatory Th_2_ responses which lead to a more severe hemorrhagic infection (11). Up-regulated expression of anti-inflammatory cytokines such as IL-6 and IL-10 facilitate Dengue virus replication and leads to disease progression (12).

Complement factor dysfunctions have been reported in patients with haemorrhagic manifestations (13). Dengue viral proteins have also been shown to reduce complement factor production. In addition, some studies have reported that in infection with Dengue, massive T cell activation results in the exacerbated production of cytokines resulting in instability in vascular endothelial cell function leading to plasma leakage in advanced stages of disease (14). Vascular leakage is a hallmark in patients with severe Dengue (15). One of the leading factors attributed to plasma leakage is T cell induced cytokine storm. Studies have shown that exacerbated cytokine secretion alters endothelial permeability resulting in severe forms of Dengue (16).

The Dengue virus primarily targets monocytes. TNFα is a monocyte derived pro-inflammatory cytokine. TNFα is known to induce reactive oxygen and nitrogen intermediates and programs for apoptotic cell death, increasing vascular permeability and ultimately leading to hemorrhage (15). Studies have shown that increased levels of TNFα are associated with increased vascular permeability in patients with Dengue. Studies have also shown that TNFα levels are raised in DWS (17). The role of TNFα in SD is still unclear. In this study, increased levels of TNFα observed in Dengue patients as compared with healthy controls are in line with above findings.

IL-6 is induced by TNFα and IL-1 (18). IL-6 has been shown to up-regulate c-reactive proteins (CRP) and secretory phospholipase A2 (sPLA2) and hence may affect endovascular permeability (18). The data for IL-6 levels in Dengue patients is variable. Some show IL-6 levels to be associated with Dengue disease severity (20). However, other studies have also reported lower levels of IL-6 in patients with shock (21). Here, we did not find any difference in IL-6 levels between healthy controls and Dengue patients in general. Lack of any difference in IL-6 levels in the general group may be due to variation between groups with differing severity of Dengue. Interestingly, IL-6 levels were lower in DWS as compared with DF where there were no warning signs. Elevated levels of IL-6 in SD as compared with patients with mild Dengue disease may indicate more pathology. Lower levels of IL-6 in DWS patients may reflect that these patients might be sampled after few days of infection when IL-6 levels tend to drop followed by an increase until when shock occurs (21–23).

Up-regulated levels of CXCL10 have previously been reported in patients infected with the Dengue virus (26). The increased CXCL10 in patients infected with Dengue virus as compared with controls observed in this study is in line with the above mentioned findings. CXCL10 has been shown to block viral entry by binding to heparin sulphate which is a co-receptor required for Dengue virus to enter the host cell (9). In this study, CXCL10 levels were found to be higher in DWS as compared with DF. Our observation that CXCL10 levels in SD were comparable with DWS or DF groups could be due to a limited sample size in the SD group.

This study had certain limitations. Samples were taken from patients at a single time point whereas the cytokine levels are reported to be dynamic and may differ at different days of illness. Also, it is ideal to sample a patient immediately after onset of the disease, but most patients seek medical advice after few days of illness. Another limitation in our study was the low sample size of patients recruited in the study which may not shed enough light on the pathogenesis of the disease.

Management of Dengue infections is primarily through supportive care. Our results show variation between IL-6 levels and increase in TNFα and CXCL10 serum levels to be associated with Dengue severity. Hence, these cytokines can be used as predictive markers of disease severity. This may assist in triaging patients as it may be difficult to assess the severity of disease at the time of diagnosis. Early identification of more advanced disease together with more proactive management may result in favorable outcomes in severe infections.

In conclusion, increased production of inflammatory mediators ultimately lead to the development of vascular permeability with poor outcome. If TNFα and CXCL10 were used as predictive biomarkers of disease progression, these biomarkers may aid in early identification of patients who are at a higher risk of more severe forms of Dengue.
